# Complex Multisystem Phenotype With Immunodeficiency Associated With *NBAS* Mutations: Reports of Three Patients and Review of the Literature

**DOI:** 10.3389/fped.2020.00577

**Published:** 2020-09-15

**Authors:** Anna Khoreva, Ekaterina Pomerantseva, Natalia Belova, Inna Povolotskaya, Fedor Konovalov, Vladimir Kaimonov, Alena Gavrina, Sergey Zimin, Dmitrii Pershin, Nataliia Davydova, Vasilii Burlakov, Ekaterina Viktorova, Anna Roppelt, Ekaterina Kalinina, Galina Novichkova, Anna Shcherbina

**Affiliations:** ^1^Dmitry Rogachev National Research and Clinical Center for Pediatric Hematology, Oncology and Immunology, Moscow, Russia; ^2^Genetics and Reproductive Medicine Center “GENETICO” Ltd., Moscow, Russia; ^3^Center of Inborn Pathology, GMS Clinic, Moscow, Russia; ^4^Veltischev Research and Clinical Institute of Pediatrics, Pirogov Russian National Research Medical University, Moscow, Russia; ^5^Genomed Ltd., Moscow, Russia; ^6^Speransky Children's Hospital, Moscow, Russia

**Keywords:** SOPH syndrome, primary immunodeficiency, osteogenesis imperfecta, liver failure, optic atrophy

## Abstract

**Objectives:** Mutations in the neuroblastoma-amplified sequence (*NBAS*) gene were originally described in patients with skeletal dysplasia or isolated liver disease of variable severity. Subsequent publications reported a more complex phenotype. Among multisystemic clinical symptoms, we were particularly interested in the immunological consequences of the *NBAS* deficiency.

**Methods:** Clinical and laboratory data of 3 patients ages 13, 6, and 5 in whom bi-allelic *NBAS* mutations had been detected via next-generation sequencing were characterized. Literature review of 23 publications describing 74 patients was performed.

**Results:** We report three Russian patients with compound heterozygous mutations of the *NBAS* gene who had combined immunodeficiency characterized by hypogammaglobulinemia, low T-cells, and near-absent B-cells, along with liver disease, skeletal dysplasia, optic-nerve atrophy, and dysmorphic features. Analysis of the data of 74 previously reported patients who carried various *NBAS* mutations demonstrated that although the most severe form of liver disease seems to require disruption of the N-terminal or middle part of *NBAS*, mutations of variable localizations in the gene have been associated with some form of liver disease, as well as immunological disorders.

**Conclusions:** *NBAS* deficiency has a broad phenotype, and referral to an immunologist should be made in order to screen for immunodeficiency.

## Introduction

Primary immunodeficiencies (PIDs) form a heterogeneous group of inherited disorders (1) that were previously considered to be very rare diseases. In recent years, the number of known PIDs has increased considerably through two lines of research: the genetic dissection of known clinical phenotypes and the discovery of new clinical phenotypes (2). PIDs are predominantly caused by mutations of the genes responsible for immune system development and various functions (1). Yet, in some PIDs, immunologic defects are part of a larger phenotype, with various multisystemic features, frequently including skeletal abnormalities. Since bone/joints defects often manifest early in life, sometimes at birth, awareness of the possible immunological and other concomitant symptoms is required to prevent life-threatening complications in these patients.

Skeletal dysplasias have been predominantly reported in association with humoral or combined immunodeficiencies (3), for instance with a large group of syndromic combined immunodeficiencies including cartilage-hair hypoplasia, Schimke immuno-osseous dysplasia, Roifman syndrome and the recently described skeletal dysplasia with neurological impairment, caused by mutations in the *exostosin-like glycosyltransferase 3* (EXTL3) gene (1, 4–6). Despite the multifaceted immune manifestations of this group of disorders, skeletal defects, such as facial dysmorphism, metaphyseal, or spondyloepiphyseal dysplasia with severe disproportionate short stature, short limbs and growth delay are usually the first symptoms that lead to specialists' referral.

Skeletal findings such as osteopenia and minimal trauma fractures have been described in another combined immunodeficiency—hyper-IgE syndrome (in both autosomal-dominant and autosomal recessive forms). Patients with autosomal dominant *signal transducer and activator of transcription 3* (*STAT3*) deficiency develop minimal trauma fractures, mostly affecting long bones and ribs; cystic changes of the bones; osteopenia, scoliosis, degenerative spine disease and craniosynostosis (7, 8). Recently, Stray-Pedersen et al. described patients with autosomal recessive hyper-IgE syndrome with *phosphoglucomutase 3 (PGM3)* deficiency who presented with severe skeletal dysplasia resembling Desbuquois dysplasia, striking skeletal abnormalities and T^−^B^−^NK^+^SCID phenotype (9).

Due to the increased use of next-generation sequencing (NGS) techniques, particularly whole-exome sequencing (WES), this list has recently been enriched by the syndrome that is caused by *neuroblastoma amplified sequence (NBAS)* gene mutations and described in patients who clinically present with severe osteogenesis imperfecta combined with immunodeficiencies and developmental delays [rev. in (10)].

*NBAS*, also known as the *neuroblastoma amplified gene (NAG)*, is a large gene comprising 52 exons and is mapped to chromosome 2p24.3, which was originally identified as frequently co-amplified with N-myc in neuroblastomas (11, 12). *NBAS* is highly expressed in cells of the connective tissues, eye, brain, and hematopoietic cells and encodes a membrane protein, a component of the Syntaxin 18 complex, that is involved in Golgi-to-endoplasmic reticulum (ER) retrograde transport and the nonsense-mediated mRNA decay (NMD) pathway. NMD also regulates normal gene expression during a wide range of physiological processes, including cell differentiation, response to stress, etc., as it can also target non-mutant transcripts (13).

Mutations in *NBAS* were initially described by Maksimova et al. (14) as a cause of short stature, optic-nerve atrophy, and the Pelger-Huët anomaly of granulocytes (SOPH) syndrome in Yakuts (OMIM 614800). This syndrome is characterized by autosomal recessive inheritance, severe postnatal growth impairment, facial dysmorphisms with senile face, small hands and feet, and normal intelligence. Subsequent case reports have described immunological defects in some patients with SOPH syndrome (10, 15–22). Recent studies demonstrated SOPH syndrome to be only a part of a clinical spectrum ranging from isolated acute liver failure to a complicated phenotype that includes skeletal dysplasia, immunological abnormalities, and the involvement of other organs (10, 16).

Herein, we describe three Russian patients with compound heterozygous mutations of the *NBAS* gene and analyze the published data in an attempt to find genotype-phenotype correlations in patients who have variable disease phenotypes.

## Methods

### Subjects

The studies involving human participants were reviewed and approved by the Independent Ethics Committee of Dmitriy Rogachev National Center for Pediatric Hematology, Oncology and Immunology (Moscow, Russia).

After informed consent peripheral blood samples were drawn from the patients and their parents. The parents consented to the publication of the photos of the patients.

### Exome Sequencing

Genomic DNA was extracted from peripheral blood. The exomes of Patients 1 and 2 and those of their parents were captured from the genomic DNA using the Agilent SureSelect Human All Exon V7 kit (Agilent Technologies, Santa Clara, CA, USA) and were sequenced (paired-end, 2 × 101 bp) using the Illumina Novaseq 6000 (Illumina Inc., San Diego, CA, USA). In the case of Patient 3, the library was prepared from Proband's genomic DNA using the TruSightOne V1.1 kit (Illumina Inc., San Diego, CA, USA) and then sequenced (paired-end, 2 × 151 bp) using the Illumina NextSeq 500 (Illumina Inc., San Diego, CA, USA).

Bioinformatic analysis of the WES data of Patients 1 and 2 and their parents was performed using a pipeline developed in-house, which included read mapping with bwa-mem, read trimming and adaptor removal using ngs-bits, local indel re-assembly using abra2, single nucleotide variation (SNV) and short indel calling using Freebayes, and variant annotation using ENSEMBL-VEP. Copy number variation (CNV) calling was performed using EXCAVATOR2 software (23). Variant prioritization was based on variant frequency in the gnomAD, variant impact predictions, known clinical pathogenicity according to ClinVar and Human Gene Mutation Database (HGMD) and family segregation data according to the ACMG criteria.

For the clinical exome data of Patient 3, another in-house pipeline was used: Read alignment to the human reference genome hg19 was performed using BWA MEM, variant calling using Freebayes, and variant annotation using the SnpEff software suite. The population frequencies in the ExAC database were used to exclude common single nucleotide polymorphisms (SNP), with subsequent variant filtering based on variant type, pathogenicity predictions, evolutionary conservation scores, and ClinVar annotations according to the ACMG classification criteria.

### Sanger Direct Sequencing

Polymerase chain reaction (PCR) Sanger sequencing was used for validation of genetic variants detected by exome sequencing. Sanger sequencing was conducted on the genomic DNA of the patients and their parents using specific primers which were designed using the online tool Primer Blast (primers sequences and PCR conditions are available upon request). Amplification products of appropriate size were identified using agarose gel electrophoresis. Products were purified with GeneJET PCR Purification Kit and then submitted to sequencing reaction using reverse primers with the ABI BigDye Terminator Cycle Sequencing Kit v. 1.1 on an ABI PRISM 3130XL Genetic Analyzer. Each read was aligned to NM_015909.3 reference sequence, and genetic variants were detected with UCSC Blat.

### Chromosomal Microarray

To validate the *NBAS* deletion detected by exome sequencing, chromosomal microarray (CMA) analysis was conducted on the genomic DNA of Patient 1 using the CytoScanHD platform (Affymetrix). The results were interpreted using the Chromosome Analysis Suite (ChAS), and ENST00000281513.5 was used as the *NBAS* reference sequence.

### Immunological Assays

The lymphocyte subsets were assessed using standard flow cytometry methods with the use of corresponding monoclonal antibodies (Becton Dickson, Franklin Lakes, NJ, USA) on the BD FACSCanto II (Becton Dickson). The levels of serum immunoglobulin were measured using the nephelometry technique on a BN ProSpec (Siemens, Berlin, Germany).

The T-cell receptor excision circle (TREC) and kappa-deleting recombination excision circles (KREC) levels were assayed in whole blood samples. Briefly, DNA was extracted from 100 μl EDTA anticoagulated whole blood by using RIBO-prep nucleic acid extraction kit (Amplisense®, Russia).

The Real-time qPCR for TRECs and KRECs was performed by using T&B PCR kit (ABV-test, Russia) on CFX 96 Real-Time PCR System (Bio Rad, USA). Amplification of ALB was used to assess correct sampling and quality of DNA extraction and to determine of TRECs and KRECs levels. The number of copies of TRECs (KRECs) was calculated per 10^5^ white blood cells, taking into account the quantity of ALB using formula: [The number of copies TRECs (KRECs)/the number of copies ALB] × 200,000. The normal/cutoff levels of TRECs and KRECs of 1,000 copies/10^5^ cells were used.

### Literature Review

We searched PubMed, EMBASE, and Scopus for studies mentioning *NBAS* defects and SOPH syndrome from their inception to August 25, 2019 and identified 23 eligible studies describing 74 patients (accounting for sample overlap).

## Results

### Clinical Phenotype

**Patient 1** (male) was born in 2006 to healthy Russian non-consanguineous parents with no relevant family history. Fetal growth delay and shortening of the upper and lower limbs were detected prenatally. The boy was delivered at term by Cesarean section and had a birth weight of 2,200 g and a length of 46 cm. He was immediately admitted to the newborn special care unit with multiple bone fractures that had occurred during delivery. The consolidation process was uneventful, but at the age of 8 months, the infant suffered a left tibia fracture as the result of a minor impact. Subsequently, he suffered multiple bone fractures and became wheelchair bound. Treatment with calcium and vitamin D did not reduce the number of fractures.

At the age of 18 months, the boy was diagnosed with bilateral partial optic nerve atrophy. Since the age of 7 months, the patient had also suffered from recurrent bronchitis and multiple episodes of pneumonia. At the age of 3 years, hypogammaglobulinemia (IgG 2.8 g/l, IgA 0.83 g/l, IgM 1.2 g/l) was noted and he was started on irregular intravenous immunoglobulin (IVIG) infusions, which somewhat reduced the number of infectious episodes.

**Figure 1:**
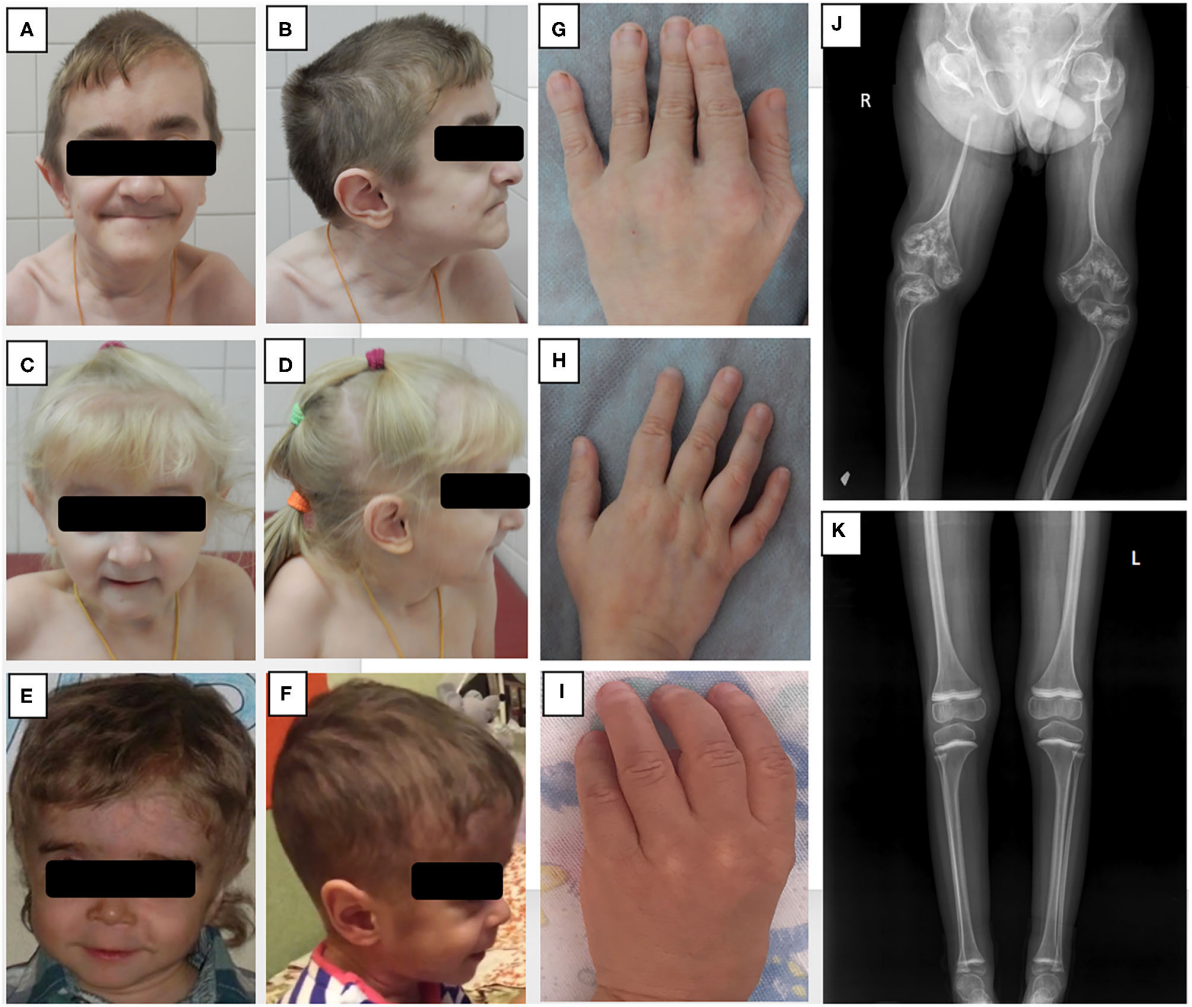
Phenotypic features and bone radiographs of three patients with *NBAS* mutations. **(A–F)** Frontal and side views of facial appearance of Patients 1, 2, and 3 at ages 13, 6, and 5, respectively. Note short neck and similar facial dysmorphism, including senile face, bilateral exophthalmos, low-set ears, narrow forehead, pointed chin, and small mouth with thin lips. **(G–I)** Brachydactyly and small hands of Patients 1, 2, and 3. **(J, K) (J)** X-ray of lower extremities of Patient 1 showing severe osteoporosis, gracile bones, signs of multiple fractures with non-union, and “popcorn” epiphyses; **(K)** X-ray of Patient 2 demonstrating mild osteoporosis with no axial deformities.

Upon examination at our tertiary center at the age of 11 years, the boy had a disproportionally short stature, muscular hypotonia, and recognizable facial features, including a senile (progeroid) appearance, a brachycephalic skull, bilateral exophthalmos, a beaked nose, thin lips, and a pointed chin (Figures 1A,B,G). His weight was 18.2 kg (<3rd centile) and his height 105 cm (<3rd centile). His intellectual development was mildly delayed. A blood workup demonstrated a normal complete blood count (CBC) with a Pelger-Huët neutrophil anomaly (Table 1). Immunologically, the number of T lymphocytes and their subpopulations was normal, yet a significant reduction in B-cells (0.11 × 10^9^/L) was demonstrated, as were low levels of IgM, IgG, and IgA (0.172, 3.05, 0.246 g/l, respectively). A computed tomography (CT) scan of his lungs showed cylindrical bronchiectasis of the right lower lobe. His blood biochemistry was normal at the time, but later a febrile infectious episode was accompanied by a mild increase in the liver enzymes aspartate aminotransferase (AST) and alanine aminotransferase (ALT) of 102 U/L (0–44 U/L) and 265 U/L (0–37 U/L), respectively (Table 1).

**Table 1:** Clinical and laboratory findings in patients with NBAS deficiency described in the current study. Patients 1 and 2 are siblings.

	**Patient 1**	**Patient 2**	**Patient 3**
Age of patient	13 years	5 years	7 years
Mutation/amino acid change	c.5741G>A, p.Arg1914His; ex. 35-47 del.	c.5741G>A, p.Arg1914His; ex. 35-47 del.	c.5741G>A, p.Arg1914His; c.1628_1629insA, p.Ser544fs
Age at onset of disease (month)	Birth	6 months	Birth
Acute liver disease episodes with elevated transaminases (during each febrile episode)	Once	Recurring	Recurring
Recurrent acute liver failure (fever-related RALF)	–	–	+
Recurrent bacterial/viral infections	Recurrent bronchitis, several episodes of pneumonia	Pharyngitis, multiple pneumonias and two episodes of infectious gastroenteritis	Multiple pneumonias, pyelonephritis
Optic nerve atrophy	Partial	Partial	Partial
Other symptoms	Intellectual delay, diabetes mellitus type 2	Intellectual delay	Pachygyria, epilepsy, feeding difficulty, severe developmental deficiency
**Skeletal findings**			
Osteogenesis imperfecta, bone fractures	+ (Multiple bone fractures)	+ (Forearm, femur fractures)	+ (Multiple bone fractures)
Bone anomalies (short stature, facial dysmorphism)	+	+	+
**Hematological findings**			
Pelger–Huët anomaly of granulocytes	+	+	+
Immune thrombocytopenia	–	–	+

*(+) presence of the symptom; (–) absence of the symptom*.

At the age of 12 years, this patient presented with an incidental finding of high blood glucose (18.3 mmol/L), and further investigation confirmed the diagnosis of diabetes type 2.

**Patient 2** is a sister of Patient 1, born at term in 2012, with a birth weight of 2,200 g and a length of 55 cm. She has had low muscle tone from birth and craniofacial and skeletal anomalies, but no fractures until 5 years of age. Since the age of 6 months, she has suffered from multiple infections, including pharyngitis, multiple pneumonias, and two episodes of infectious gastroenteritis. Laboratory evaluation at the age of 1 year revealed elevated levels of the liver enzymes AST [86.8 U/L (0–44 U/L)] and ALT [338 U/L (0–37 U/L)], yet infectious causes of hepatitis were excluded. At the age of 4 years, hypogammaglobulinemia (IgG 2.7 g/L, IgA 0.26 g/L, and IgM 0.5 g/L) was found and she was also started on immunoglobulin replacement therapy (Table 2).

**Table 2:** Immunological findings in patients with NBAS deficiency described in the current study.

**Parameter**	**Patient 1**	**Patient 2**	**Patient 3**	**Reference range**
	Lymphocyte subset analysis
Lymphocyte count, × 10^9^/L	5.38	2.71	1.17	2.3–5.4
**T cells**				
CD3+, × 10^9^/L	5.2	2.43	1.05	1.4–3.7
CD3+ CD4+, × 10^9^/L	0.9	1.43	0.17	0.7–2.2
CD3+ CD8+, × 10^9^/L	4.18	0.61	0.87	0.49–1.3
CD4+CD45RA+ naïve T cells	0.56	1.04	0.05	0.43–1.50
CD4+CD45RO+ memory T cells	0.38	0.25	0.05	0.22–0.66
CD8+CD45RA+ naïve T cells	2.23	0.44	0.02	0.38–1.10
CD8+CD45RO+ memory T cells	1.45	0.06	0.001	0.09–0.44
**B cells**				
CD19+, × 10^9^/L	0.11	0.24	0.07	0.4–1.7
CD19+CD27-IgD+ naive, %	1.58	6.5	23.6	54–88
CD19+CD27-IgD+ naive, × 10^9^/L	0.08	0.17	0.01	0.28–1.33
CD19+CD27+IgD+ pre-switch memory B cells, %	0.32	0.43	37.2	2.7–19.8
CD19+CD27+IgD+ pre-switch memory B cells, × 10^9^/L	0.017	0.012	0.02	0.02–0.18
CD19+CD27+IgD- post-switch memory B cells, %	0.13	0.87	12.16	4.7–21.2
CD19+CD27+IgD- post-switch memory B cells, × 10^9^/L	0.007	0.02	0.009	0.04–0.14
**NK cells**				
CD3– CD16+CD56+, × 10^9^/L	0.16	0.04	0.05	0.13–0.72
**TREC/KREC**
TREC, copy numbers per 10^5^ leukocytes	910	3,500	44	470–4,100
KREC, copy numbers per 10^5^ leukocytes	120	190	1	780–7,700
	Serum immunoglobulins^*^
IgG (g/L)	2.8	2.7	1.6	6.8–15.4
IgM (g/L)	1.2	0.5	0.13	0.8–1.6
IgA (g/L)	0.83	0.26	0.05	0.3-1.5

*Reference range values (24, 25)*.

* *Igg serum concentration before IVIG substitution*.

Upon examination at our tertiary center at the age of 5 years, she had facial dysmorphic features similar to those of her brother, a disproportionately short stature (93 cm, −3.5 SD), partial atrophy of the optic nerve and cognitive difficulties (Figures 1C,D,H). Her CBC and immunological workup were very similar to those of her brother (Table 2), and she was noted to have elevated liver enzymes [AST of 343 U/L, ALT of 250 U/L, and lactate dehydrogenase (LDH) of 483 U/L]. An abdominal ultrasound revealed diffuse liver parenchymal heterogeneity. A needle liver biopsy showed minimal changes, with no signs of fibrosis. In terms of the skeletal phenotype, the patient had experienced forearm and femur fractures at 5 and 6 years of age, respectively.

Whole-exome quartet sequencing of the DNA samples from Patients 1 and 2 and their parents revealed two genetic variants in *NBAS* gene. Both patients carried a previously reported c.5741G>A (p.Arg1914His) “Yakut” mutation located in exon 45 in hemizygous state inherited from their mother and a large novel 120 kbp deletion of the exons 35–47, inherited from their father (Supplementary Figure 1). The deletion was confirmed using CMA and its molecular karyotype was established: arr[hg19] 2p24.3(15370717_15492334)x1 (Supplementary Figure 2).

The patients were treated with regular IVIG infusions at doses of 0.6 g/kg/month, with trough IgG levels reaching target levels of >6 g/l. The treatment was effective in both siblings, significantly reducing the number of infections. The patients were also referred to the Center for Inborn Pathology (GMS Clinic) for further treatment of osteoporosis. In Patient 1 X-rays found very gracile bones with extremely thinned femur diaphysis, severe osteoporosis, bowing deformities, and non-union of the right radius and left femur (Figure 1J). A dual energy X-ray absorptiometry (DEXA) scan revealed severe osteoporosis with a *z*-score of L1–L4 −3.8. On X-ray of Patient 2 osteoporosis without axial deformities was found (Figure 1K), while DEXA scan revealed severe decrease of lumbar bone density with total L1–L4 *Z*-score −3.4. Treatment included bisphosphonates (pamidronate 1 mg/kg every 4 months), physical therapy, and orthopedic recommendations.

**Patient 3** (male) is a second child of healthy unrelated Russian parents without relevant family history. He was born in 2011 with a birth length of 50 cm and a weight of 2,780 g. After birth, the infant was treated in the neonatal intensive care unit for pneumonia and multiple bone fractures (both clavicles, a femur, and the 5–7th ribs). A diagnosis of osteogenesis imperfecta was suspected.

At the age of 3 months, he was diagnosed with immune thrombocytopenia and successfully treated with steroids. Since the age of 4 months, he has had multiple episodes of pneumonia and pyelonephritis. Since 11 months, varying degrees of liver enzyme elevation have been noted (AST of 33–400 U/L, ALT of 43–450 U/L).

At the age of 3 years, he developed *Pseudomonas aeruginosa* sepsis accompanied by febrile liver crises, vomiting, seizures, and asphyxia, requiring admission to a pediatric intensive care unit for mechanical ventilation and subsequent severe brain damage.

Examination at our center at the age of 5 years revealed severe generalized developmental delay, typical dysmorphic features (macrocephaly, low-set ears, sparse teeth, prominent forehead), contractures of the elbow, wrist, and knee joints, marked muscular hypotonia, partial atrophy of the optic nerve, and hepatomegaly (Figures 1E,F,I). His poor feeding due to the brain damage/developmental impairment necessitated placement of a gastrostomy tube. Laboratory tests showed elevated levels of AST (397 U/L), ALT (457 IU/L), and serum ferritin (1,183 ng/ml, normal range: 20–120) and hyperammonemia (116 μmol/L). Immunological examination revealed significantly reduced levels of serum immunoglobulins (IgG 1.6 g/L, IgA 0.05 g/L, IgM 0.13 g/L). Immunophenotyping identified a reduced number of T-cells (in contrast to Patients 1 and 2) and a low number of B-cells (Table 2).

Clinical exome sequencing revealed two heterozygous variants in the *NBAS* gene: c.5741G>A, (p.Arg1914His), and c.1628_1629insA (p.Ser544fs).

Treatment with IVIG infusions at a dose of 0.4 g/kg/month and prophylactic co-trimoxazole was started at the age of 5 years, which decreased the frequency of invasive bacterial infections. However, the patient is severely disabled due to the encephalopathy.

### Literature Review

**Figure 2:**
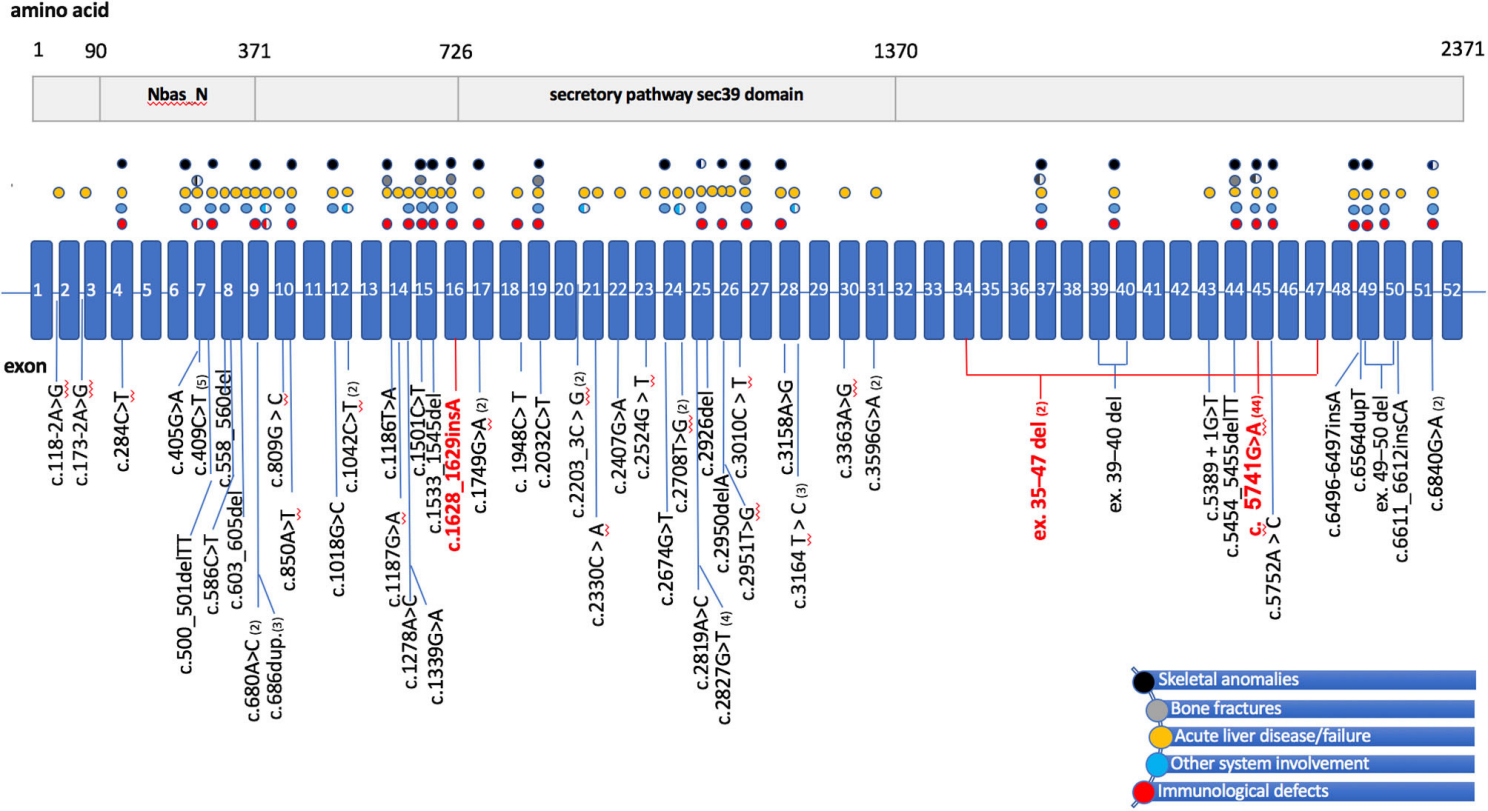
Schematic representation of the *NBAS* gene/protein with mutations of patients in literature and their main symptoms. **(Top)** A schematic representation of the NBAS protein, showing the two predicted functional domains: neuroblastoma-amplified sequence, N-terminal (NBAS_N) and secretory pathway protein Sec39 (Sec39). **(Bottom)** The *NBAS* gene structure, with exons (numbered 1–52) indicated by boxes and introns by a straight blue line. Positions of mutations are displayed below the gene diagram. Mutations of patients in the current study are highlighted red, mutations reported to date are shown in black, and parentheses include the numbers of cases described. Colored circles above the gene scheme refer to patients' clinical features and correspond to the mutations below the gene diagram, with circles colored as follows: black for skeletal anomalies, including facial dysmorphism, short stature, skeletal dysplasia; gray for bone fractures; yellow for acute liver failure or acute liver disease triggered by fever; blue for involvement of other systems, including Pelger-Huët anomaly and optic nerve atrophy; red for immunodeficiency. Partially filled circles indicate that the clinical features have been detected in only some of the individuals with the particular mutation. del, deletion; dup, duplication; ins, insertion.

In all, we identified reports of 77 patients with *NBAS* deficiency (including the 3 patients described above), who carried 52 different mutations in the homozygous or compound heterozygous state. These included 34 Yakut patients from the original report of Maksimova et al., who were homozygous for the c.5741 G>A mutation. This mutation in a compound-heterozygous state has been reported in 7 additional patients (17, 19, 21, 26). Other mutations included missense, nonsense, and splice-site variants that affected most exons and some exon-intronic junctions. Two large deletions involving exons 39–40 and 49–50 have been also reported (Figure 2).

The mean age of the patients was 9.9 ± 7.3 years, the age on disease onset was 40 months ± 1.1 year. Details of clinical presentations and immunological phenotypes are summarized (Supplementary Table 1).

Various degrees of liver disease have been described in 39 patients, recurrent acute liver failure (RALF) being the most frequent and severe. This frequently occurs in early childhood and leads to significant morbidity and mortality. Although some degree of liver disease has been associated with most *NBAS* variants, the mutations associated with severe liver phenotype were either loss-of-function or missense mutations predominantly located in the N-terminal and in the middle part of the *NBAS* gene (c.409C>T identified in 5 patients: c.680A>C;1749G>A, c.809G>C;2926del, c.1018G>C;2674G>T, c.2819A>C, and c.2819A>C) (15, 18, 27–31).

The next most frequent feature of *NBAS* deficiency was bone anomalies, which were reported in more than 90% of the patients, including severe osteogenesis imperfecta in 8 patients.

Immunological abnormalities leading to recurrent ear and upper and lower respiratory-tract infections were reported in 15 patients (10, 15–22, current study), although this number could be larger, since some studies did not describe patients' infectious and immunological status in detail. Immunological data in available patients demonstrated an impaired humoral immunity with lack of response to vaccinal antigens, hypogammaglobulinemia, low B cells and KRECs (10, 15–17, 20–22). T-cell deficiency with low CD3+CD8+ lymphocytes and severe infections was described in two patients previously (10, 18) and in one of the patients in the current study. Progressive reduction in NK cells numbers has been described by *Garcia* Segarra et al. (15) and then by Ricci et al. (10). As Figure 2 demonstrates, the patients who had immunodeficiency and bone disease had mutations distributed along the gene, with no particular domain predominance (10, 14–22, 27–35), current study).

## Discussion

The complexity and high variability of the disease phenotype, observed even within the same family, in which one of the siblings has more-advanced bone involvement (a clinical presentation of severe osteogenesis imperfecta, requiring bisphosphonates treatment) and his sister has milder skeletal abnormalities but more severe liver disease and vision damage. The case of the third, unrelated patient also provides evidence of the devastating consequences of the disease.

The variability of the symptoms might be explained by the putative functional aspects of the *NBAS* protein. Although its exact role in the cells has not been delineated, *NBAS* is known to be a subunit of the Syntaxin 18 complex, a soluble NSF-attachment protein (SNAP) receptor localized in the ER and involved in retrograde transport from the Golgi complex to the ER in human cells (36). Impairment of this transport has been demonstrated in *NBAS*-mutated cell lines, resulting in ER stress (37). This might explain liver disease in the setting of the high-energy catabolic state during a febrile episode. Liver disease in many of the patients described was characterized by recurrent elevated transaminases, hypertriglyceridemia, hyperbilirubinemia, and elevated ferritin. The most severe form of liver disease, RALF, seemed to require disruption of the N-terminal or middle part of the *NBAS*, where most of the functional domains are located. Yet, our literature analysis showed that both N-terminal and C-terminal mutations have been associated with some form of liver disease. Even though patients with homozygous “Yakut” c.5741G>A mutation do not demonstrate liver disease, other patients with this mutation in the compound heterozygous state, including patients from the current study, showed signs of liver disease. Syntaxin 18 forms a complex with multiple proteins, including p31 and ZW10-RINT-1 (38). Aoki et al. demonstrated that the N-terminal part of *NBAS* is required for p31 binding, whereas ZW10-RINT-1 binds the *NBAS* C-terminal part. Thus, *NBAS* serves as a link between p31 and ZW10-RINT-1, and both its N-terminal and C-terminal domains are required for this. Therefore, it is expected that mutations of the NBAS N-terminus, as well as C-terminus affect its function and might cause liver disease in patients.

Another essential function of the NBAS protein is participation in the NMD pathway in human cells. NMD is thought to prevent the translation of potentially disease-causing truncated proteins via rapid mRNA degradation when premature termination codons (PTC) are formed (39). Therefore, NMD plays an important role in modulating the phenotypic outcome of genetic disorders that are caused by mutations leading to PTC. However, it has become evident in recent years that NMD is also important for physiological control of the expression of naturally occurring transcripts in developing cells and in cells responding to various stimuli (40). Analysis of the RNA sequencing data in cells in which NMD is disrupted has shown that it has a widespread effect on gene expression and has led to the identification of multiple putative NMD target mRNAs (41). A large number of targets were shown to be downregulated in response to NBAS depletion. Among them were several genes that play a role in bone development and cholesterol biosynthesis. Defects in these processes can explain bone defects in patients with *NBAS* deficiency. For instance, depletion of NBAS led to a significant upregulation of matrix G1a protein (MGP), which regulates bone formation (42). The NMD response has been shown to be variable among different cell types (43) and even among individuals (44), which might explain significant symptom variability in *NBAS* deficiency. In our analysis, the reported cases showed no correlation of the mutation types and localization with particular symptoms or disease severity, leading to the conclusion that epigenetic rather than genetic mechanisms are at play here.

Among the multisystemic clinical symptoms previously described in patients with *NBAS* deficiency, we were particularly interested in the immunological consequences of the defect. *NBAS* was originally shown to be expressed in ganglion cells, squamous epidermal cells, and in leukocytes, suggesting the crucial functions of these cell types (14). The first association of the *NBAS* gene with immunodeficiency was established by Garcia Segarra et al. in two patients who had *NBAS* mutations and suffered from recurrent viral and bacterial infections, including pneumonia and middle ear infections, and who were reported to have hypogammaglobulinemia, reduced NK cells, and abnormal antibody production in response to vaccinations. Our patients' immunological workups demonstrated normal or low numbers of T-cells, the near-absence of B-cells, and markedly reduced serum levels of IgA, IgG, and IgM. The decrease in KRECs observed in our patients emphasizes the defect in B-cell production. The recurrent sinopulmonary infections experienced by our patient and others in the literature are consistent with a defect in humoral immunity. However, a susceptibility to viral infections, most commonly herpes simplex, varicella-zoster, and Epstein-Barr, has also been observed (15, 19). During their development, lymphocytes undergo V(D)J genomic rearrangements to assemble immunoglobulins and B- and T-cell receptor genes. Two-thirds of these rearrangements yield unproductive gene products harboring PTCs, whose clearance requires NMD (45). Therefore, NMD dysfunction might explain a lack of B-cells and sometimes T-cells which likely leads to hypogammaglobulinemia in patients with NBAS mutations. In addition, this could also explain the lack of specific correlation of phenotype with the location of a defect within the gene. However, another explanation for the patients' viral susceptibility may lie in the fact that NMD has been shown to also serve as a barrier to viral replication, suppressing expression of viral proteins and limiting viral titer in host cells (46).

## Conclusion

According to multisystem phenotype, *NBAS*-deficient patients are followed by various specialists, it is important that these patients are referred to immunologists early to be screened for immunodeficiencies. We suspect that the frequency of immunological defects in *NBAS* deficiency has been underestimated and may be higher than it appears from the literature. There is no standardized treatment for *NBAS*-deficient patients. Therefore, a multidisciplinary approach is crucial to diagnosing and managing them. Severe osteoporosis with bone deformities may be one of the dominant features and requires treatment using bisphosphonates. Developmental delays and serious vision damage significantly affect the quality of life of these patients. In addition, immunodeficiency seems to be an under-recognized feature of *NBAS* deficiency and requires further investigation. All patients with hypogammaglobulinemia should receive regular infusions of immunoglobulins to reduce the frequency of sinopulmonary infections.

Recent research has aimed to develop therapeutic modalities for NMD. There is experimental evidence that therapeutic decrease in the NMD response might benefit those with some genetic disorders and certain types of tumors (47–49). Studying *NBAS*-deficient patients may provide a better understanding of the possible side effects of such treatment. In addition, information regarding the NMD modulation options can be used to develop drugs that increase the NMD response, which might be therapeutic in *NBAS* deficiency.

## Data Availability Statement

The raw data supporting the conclusions of this article will be made available by the authors, without undue reservation.

## Ethics Statement

The studies involving human participants were reviewed and approved by the Independent Ethics Committee of Dmitriy Rogachev National Center for Pediatric Hematology, Oncology and Immunology (Moscow, Russia). After informed consent peripheral blood samples were drawn from the patients and their parents. The parents consented to the publication of the photos of the patients.

## Author Contributions

AS drafted the initial manuscript, reviewed and revised the manuscript for important intellectual content, and gave final approval of the version to be submitted. GN provided critical feedback and helped to shape the analysis and manuscript. AK and NB drafted the initial manuscript, collected the data, reviewed, and revised the manuscript. EP, IP, FK, and VK supervised the genetic tests and took part in drafting the article. DP, ND, and EK supervised the immunological assays and reviewed the manuscript. AG, SZ, VB, EV, and AR collected data, participate in drafting the article, and reviewed the manuscript. All authors contributed equally to this work, approved the final manuscript as submitted, and agree to be accountable for all aspects of the work.

## Conflict of Interest

EP, IP, and VK were employed by the company Genetics and Reproductive Medicine Center “GENETICO” Ltd., Moscow. FK was employed by the company Genomed Ltd., Moscow, Russia. The remaining authors declare that the research was conducted in the absence of any commercial or financial relationships that could be construed as a potential conflict of interest.

## Supplementary Material

Supplementary Figure 1 Sanger sequencing results, confirming mutations in patients 1–2. *NBAS* c.5741G>A genetic variant in patients 1 and 2 and their parents (reverse chain).

Supplementary Figure 2 Patient 1 CMA results (CytoScanTM HD Array). 122 kb deletion arr[GRCh37] 2p24.3(15370718_15492334)x1, involving exons 35–47 of the *NBAS* gene is highlighted in red.

Supplementary Table 1 Literature review of 77 patients with *NBAS* deficiency.

## Abbreviations

ALT: alanine aminotransferase
ALF: acute liver failure
AST: aspartate aminotransferase
ER: endoplasmic reticulum
IVIG: intravenous immunoglobulin
KREC: kappa-deleting recombination excision circles
NBAS: neuroblastoma-amplified sequence
NAG: neuroblastoma amplified gene
NMD: nonsense-mediated mRNA decay
PID: primary immunodeficiency
PTC: premature termination codons
RALF: recurrent acute liver failure
SOPH, short stature, optic nerve atrophy: and Pelger–Huët anomaly of granulocytes
TREC: T-cell receptor excision circle.
