# Supplementary figures and images for: Complex Multisystem Phenotype With Immunodeficiency Associated With NBAS Mutations: Reports of Three Patients and Review of the Literature
Source: Front Pediatr. 2020 Sep 15;8:577. doi: 10.3389/fped.2020.00577 (PMC7522312; doi:10.3389/fped.2020.00577)

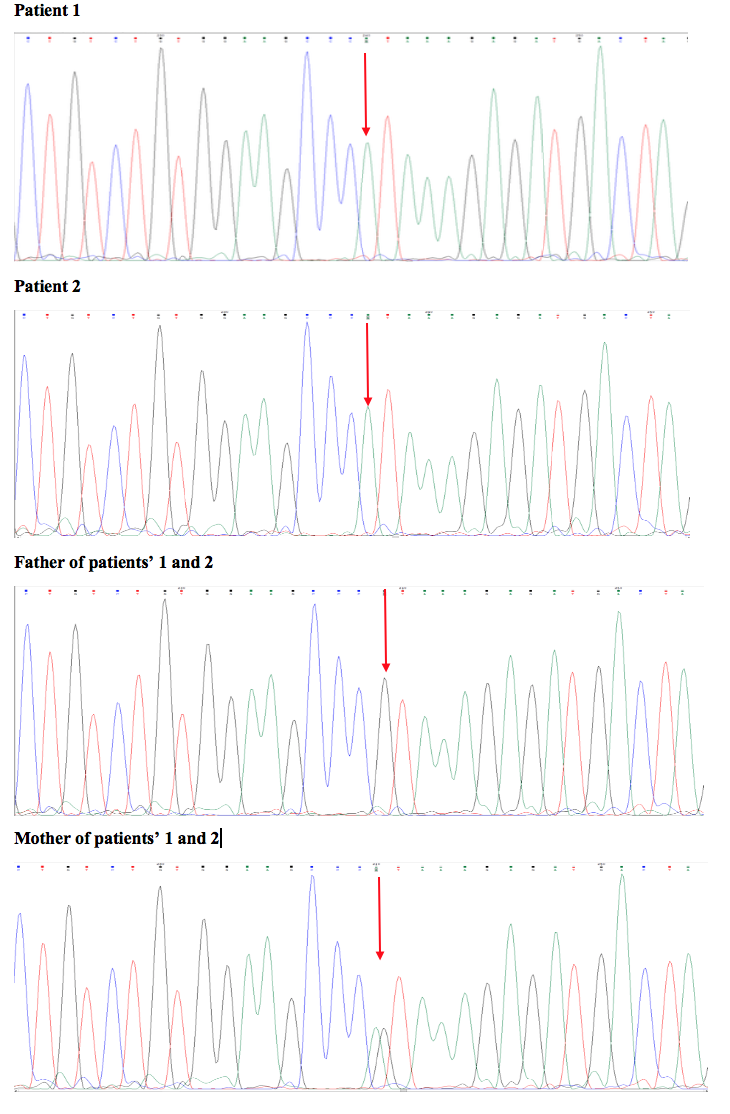

Supplement: Supplementary Figure 1 — Sanger sequencing results, confirming mutations in patients 1–2. NBAS c.5741G>A genetic variant in patients 1 and 2 and their parents (reverse chain). [file Image_1.PNG]

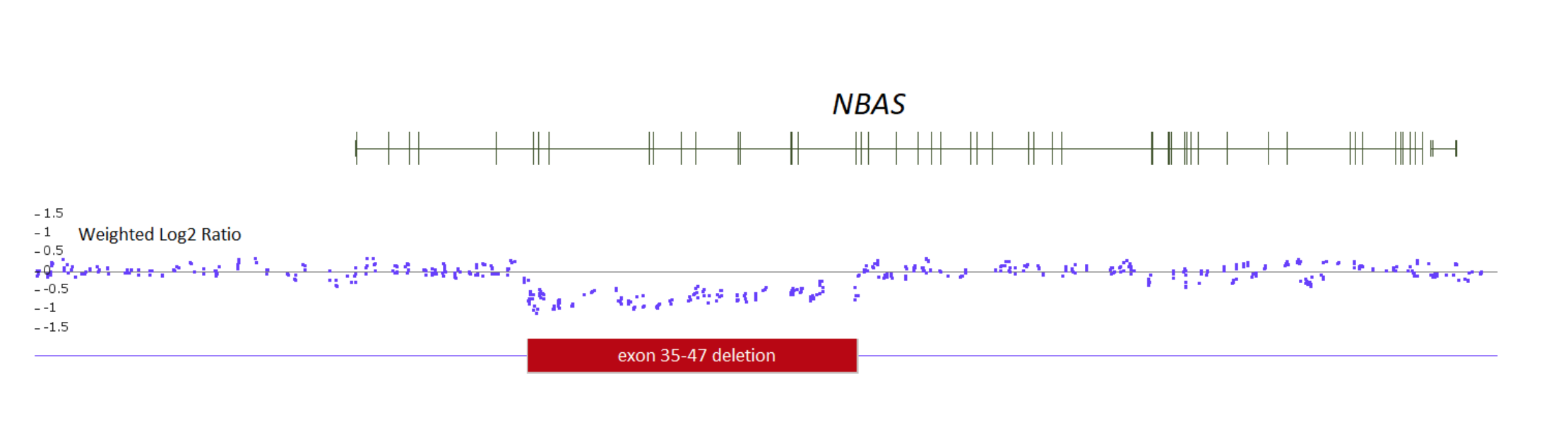

Supplement: Supplementary Figure 2 — Patient 1 CMA results (CytoScanTM HD Array). 122 kb deletion arr[GRCh37] 2p24.3(15370718_15492334)x1, involving exons 35–47 of the NBAS gene is highlighted in red. [file Image_2.PNG]
